# Mesoporous Silica MCM-41 from Fly Ash as a Support of Bimetallic Cu/Mn Catalysts for Toluene Combustion

**DOI:** 10.3390/ma17030653

**Published:** 2024-01-29

**Authors:** Jakub Mokrzycki, Monika Fedyna, Dorota Duraczyńska, Mateusz Marzec, Rafał Panek, Wojciech Franus, Tomasz Bajda, Robert Karcz

**Affiliations:** 1 Faculty of Energy and Fuels, AGH University of Krakow, Mickiewicza 30 Av., 30-059 Krakow, Poland; 2 Faculty of Chemistry, Jagiellonian University, Gronostajowa 2, 30-387 Krakow, Poland; monika.fedyna@uj.edu.pl; 3 Jerzy Haber Institute of Catalysis and Surface Chemistry, Polish Academy of Sciences, Niezapominajek 8, 30-239 Krakow, Poland; dorota.duraczynska@ikifp.edu.pl (D.D.); robert.karcz@ikifp.edu.pl (R.K.); 4 Academic Centre for Materials and Nanotechnology, AGH University of Krakow, Mickiewicza 30 Av., 30-059 Krakow, Poland; marzecm@agh.edu.pl; 5 Department of Construction Materials Engineering and Geoengineering, Civil Engineering and Architecture Faculty, Lublin University of Technology, Nadbystrzycka 40, 20-618 Lublin, Poland; r.panek@pollub.pl (R.P.); w.franus@pollub.pl (W.F.); 6 Faculty of Geology, Geophysics and Environmental Protection, AGH University of Krakow, Mickiewicza 30 Av., 30-059 Krakow, Poland; bajda@agh.edu.pl

**Keywords:** MCM-41, catalysts, toluene combustion, fly ash

## Abstract

The main outcome of this research was to demonstrate the opportunity to obtain a stable and well-ordered structure of MCM-41 synthesized from fly ash. A series of bimetallic (Cu/Mn) catalysts supported at MCM-41 were prepared via grinding method and investigated in catalytic toluene combustion reaction to show the material’s potential application. It was proved, that the Cu/Mn ratio had a crucial effect on the catalytic activity of prepared materials. The best catalytic performance was achieved with sample Cu/Mn(2.5/2.5), for which the temperature of 50% toluene conversion was found to be 300 °C. This value remains in line with the literature reports, for which comparable catalytic activity was attained for 3-fold higher metal loadings. Time-on-stream experiment proved the thermal stability of the investigated catalyst Cu/Mn(2.5/2.5). The obtained results bring a valuable background in the field of fly ash utilization, where fly ash-derived MCM-41 can be considered as efficient and stable support for dispersion of active phase for catalyst preparation.

## 1. Introduction

Constantly growing global industrial production and transport are causing enormous emissions of volatile organic compounds (VOCs). VOCs are a broad class of low molecular weight chemicals containing carbon. Great attention is paid inter alia to the so-called BTEX (benzene, toluene, ethylbenzene, and o-, m-, p-xylene), which are identified as toxic volatile compounds [1]. Various methods of their removal from the aqueous and gas phase are constantly investigated. The most frequently used methods involve: adsorption (liquid, gas phase), membrane separation (gas phase), wet gas scrubbing (gas phase), condensation (gas phase) and catalytic conversion (gas phase). Among the abovementioned, catalytic conversion can be considered as the most economical because BTEX can be converted into new value-added raw materials or can be directly utilized. 

Catalytic combustion of toluene as a model VOC has been investigated over various catalytic systems, i.e., mixed oxides CuO/Mn_2_O_3_ [2], cobalt oxides [3,4], spinel oxides [5], Pt-supported catalysts at mesoporous silica (MCM-48) [6], and manganese oxides (MnO_x_) supported on zeolites (HZSM-5) [7]. Attention is paid to catalysts supported at mesoporous silicates, as they are mechanically, thermally and chemically stable, whereas manganese oxides are reported to be highly active. Mobil composition of matter (MCM-41) is an amorphous, mesoporous silicate molecular sieve containing highly regular pores of average diameter from 1.5 to 10 nm. MCM-41 is characterized by high mechanical, chemical and thermal stability and high specific surface area (S_BET_) up to 1000 m^2^ g^−1^ [8]. Due to its unique properties, MCM-41 has gained the attention of researchers and has been widely investigated in various fields i.e., catalysis, adsorption, gas storage and separation. A great opportunity for environmental protection is the synthesis of MCM-41 from waste materials like fly ashes [9]. In fact, fly ashes were already reported to be CO_2_ sequestration agents [10], as well as effective raw materials for the synthesis of zeolites [11,12], nutrients adsorption from aqueous solutions, and PAH-contaminated (polycyclic aromatic hydrocarbons-contaminated) soil restoration agents [13]. 

Fu et al. [14] investigated bimetallic Pt/Pd catalysts supported at MCM-41 in the reaction of catalytic combustion of toluene. The evaluated metal loadings varied from 0 to 0.3 wt.%. The monometallic system containing 0.3 wt.% Pd was the least active catalyst among the investigated series. Toluene conversion of 100% was obtained at 220 °C, whereas for catalysts containing Pt (both in monometallic and bimetallic system) the temperature was lowered by 20 °C. The exception was the bimetallic system containing 0.2 wt.% Pt and 0.1 wt.% Pd, for which the 100% toluene conversion was obtained at 180 °C. The authors claimed, that the reason for such a phenomenon was related to the type of Pt species at the catalyst surface, for which Pt(0) was the most active. A synergic effect of combining two noble metals was hence confirmed. Noble metals are however expensive, and for this reason there is a constant search for non-noble metal catalysts. The d-block metal oxides and mixed metal oxides (spinel) were found to possess sufficient activities in toluene combustion. They are much cheaper than noble metals and are characterized by a greater resistance to poisoning; however, their durability is limited, when compared with supported catalysts [15]. Attention is paid to manganese oxides (MnO_x_) and copper oxides (CuO_x_). Bimetallic Cu/Mn catalysts supported on MCM-41 were investigated by Li et al. [16]. The investigated metal doses were 15.60 wt.% of CuO and 0.72 wt.% of MnO. Also single-metal catalysts were prepared with Cu or Mn loading of 15.8 and 15.2 wt.%, respectively. It was reported, that the greatest catalytic activity was obtained for the bimetallic system; the temperature of 90% toluene conversion (T_90_) was about 325 °C, whereas for Cu and Mn single-metal catalysts this value shifted toward higher temperatures (above 475 °C). The authors also underlined the positive effect of supporting the metal phase at mesoporous materials instead of microporous zeolites, as the coke resistance was found to be much greater for MCM-41 support. 

An important factor that can further affect the results is the method of the active phase introduction. The most common methods involve wet or dry impregnation. However, it was reported, that the solid-state grinding method can allow introducing well-dispersed active phase into the mesoporous materials [17]. Due to the simplicity of the method, and improvement of the catalyst’s activity (also reflected in a stronger metal-support interaction), such treatment is gaining attention [18]. 

The present study aimed to show the opportunity to use MCM-41 from fly ash as an efficient catalyst of toluene combustion. In this research, a bimetallic and monometallic catalytic systems were investigated, where Cu and/or Mn were introduced into the MCM-41 via the grinding method. The metal loadings of Cu/Mn were: 0/5; 1/4; 2.5/2.5; 4/1; and 5/0 wt.%, respectively. The maximum metal loading of 5 wt.% was selected to allow lower metal utility for catalyst preparation. Materials were characterized in detail by means of XRD, nitrogen adsorption-desorption, XRF, XPS, SEM-EDS, Raman spectra, and oxygen temperature programmed desorption (O_2_-TPD). 

## 2. Materials and Methods

### 2.1. MCM-41 Synthesis from Fly Ash 

Mesoporous silica type of MCM-41 was synthesized by a templating method using the waste solution after zeolite synthesis from fly ash, as was described in previous works [9]. Briefly, 5 g of CTAB (Sigma Aldrich, Burlington, MA, USA) was added to 100 mL of distilled water and stirred until dissolved. Then 4M sulfuric acid (Sigma Aldrich, Burlington, MA, USA) was added and stirred for several minutes while monitoring the pH which was below pH = 1. The post-reaction filtrate (a silica precursor) was added to the CTAB solution until pH = 11. Then the whole mixture was heated to 40 °C and stirred for 1 h. Next, the pH was checked and, if necessary, adjusted to pH = 11. The obtained solution was poured into a Teflon reactor and placed in a dryer at 100 °C for 24 h. After this time, the solution was cooled and filtrated. The obtained precipitate was washed with distilled water to remove excess amounts of surfactant. In the last stage, to remove the surfactant template, the obtained product was placed in a muffle furnace (Czylok, Jastrzębie-Zdrój, Poland) at 550 °C (at a heating rate of 1 °C min^−1^) and calcined for 6 h in an air atmosphere.

### 2.2. Catalysts Preparation

MCM-41 (about 2 g) was mixed in a mortar for 10 min with proper amounts of Cu(NO_3_)_2_·3H_2_O (Sigma Aldrich, Burlington, MA, USA) and/or Mn(NO_3_)_2_·4H_2_O (Sigma Aldrich, Burlington, MA, USA), to obtain metal loading of Cu/Mn of 5/0, 4/1, 2.5/2.5, 1/4, or 0/5 wt.% at MCM-41. Next, the samples were placed in an electric oven FCF8 (Czylok, Jastrzębie-Zdrój, Poland) for calcination at 550 °C for 6 h. After cooling to room temperature, samples were collected and stored for further analysis. The obtained materials were denoted as follows: Cu/Mn(5/0), Cu/Mn(4/1), Cu/Mn(2.5/2.5), Cu/Mn(1/4), Cu/Mn(0/5).

### 2.3. Materials Characterization

#### 2.3.1. X-ray Diffraction (XRD)

Phase composition was investigated using the XRD method. The measurements were conducted in a scan range of 2θ varying from 1 to 70° with a 0.05° min^−1^ step. X′pert PROMPD diffractometer (CuKα radiation and λ = 0.15418 nm) with a PW3050/60 goniometer and a Rigaku SmartLab diffractometer (Tokyo, Japan) were employed for analysis. 

#### 2.3.2. Nitrogen Adsorption–Desorption at −196 °C

Nitrogen adsorption-desorption isotherms were collected at −196 °C using the ASAP 2020 instrument (Micromeritics, Norcross, GA, USA). Prior to the analysis samples were degassed overnight at 300 °C. The total pore volume (V_t_) and average pore diameter (D_p_) were obtained from the BJH method.

#### 2.3.3. X-ray Fluorescence Spectroscopy (XRF)

The gain a better knowledge about the chemical composition of supported MCM-41 materials, the samples were subjected to X-ray fluorescence (XRF) measurement using an ARL QUANT’X spectrometer (Thermo Scientific, Waltham, MA, USA) with a rhodium anode (4–50 kV in 1 kV increments) and beryllium windows. A 1 mm beam and a 3.5 mm Si(Li) drifted crystal detector with Peltier cooling (at about −88 °C) were used for all measurements. Quantitative analysis of elements within the catalysts was performed using UniQuant software (Version 3, Thermo Fisher, West Palm Beach, FL, USA) and metallic calibration standards.

#### 2.3.4. Scanning Electron Microscopy (SEM) with Energy Dispersive X-ray Analysis (EDS)

Aiming to investigate the surface of the materials, catalysts were investigated by means of SEM-EDS. Prior to imaging, samples were coated with a thin layer of chromium using a K575X Turbo Sputter Coater (Quorum Emitech, South Stour Avenue, Ashford, UK)—deposited film thickness was 20 nm. Images were taken using the JEOL JSM 7500 F (JEOL, Tokyo, Japan) instrument, equipped with the energy-dispersive X-ray spectroscopy (EDS) system (Oxford Instruments Aztec LIVE Lite, High Wycombe, UK).

#### 2.3.5. Raman Spectra

Raman spectra were recorded at room temperature in the range of 150–1200 cm^−1^ with a Renishaw InVia spectrometer (Renishaw, Wootton-under-Edge, UK) using the 788 nm wavelength.

#### 2.3.6. X-ray Photoelectron Spectroscopy (XPS)

The XPS analyses were carried out in a PHI VersaProbeII Scanning XPS system using monochromatic Al Kα (1486.6 eV) X-rays focused to a 100 µm spot and scanned over the area of 400 µm × 400 µm. The photoelectron take-off angle was 45° and the pass energy in the analyzer was set to 117.50 eV (0.5 eV step) for survey scans and 46.95 eV (0.1 eV step) to obtain high energy resolution spectra for the C 1s, O 1s, Si 2p, Mn 2p and Cu 2p regions. A dual beam charge compensation with 7 eV Ar^+^ ions and 1 eV electrons was used to maintain a constant sample surface potential regardless of the sample conductivity. All XPS spectra were charge referenced to the unfunctionalized, saturated carbon (C-C) C 1 s peak at 285.0 eV. The operating pressure in the analytical chamber was less than 3 × 10^−9^ mbar. Deconvolution of spectra was carried out using PHI MultiPak software (v.9.9.3). Spectrum background was subtracted using the Shirley method.

### 2.4. Catalytic Tests

#### 2.4.1. Toluene Combustion

Toluene catalytic combustion was performed in a fixed-bed quartz reactor. The inner diameter was 10 mm and the catalyst loading was 0.5 cm^3^ of prepared granules. Before the test, the catalyst was pressed into pellets, grounded and sieved to obtain a fraction 0.3–0.5 mm. The temperature range of the catalytic tests was from 150 to 450 °C (up to 400 °C for parent MCM-41 material). Prior to the catalytic test, the materials were placed in the reactor and heated for 1 h in the flow of air up to 300 °C. Toluene (500 ppm in air) was fed into the reactor at 10,000 GHSV h^−1^ (total flow of 84 mL min^−1^). After reaching each reaction temperature, the reactor was stabilized for 30 min before analysis of samples from the gas stream. Samples were collected using 1000 μL Hamilton gastight syringe at the reactor inlet and outlet. Gas composition was analyzed using Perkin-Elmer Clarus 500 GC system (Perkin-Elmer, Shelton, CT, USA) equipped with Elite-1 column (30 m, 0.32 mm ID, 3 μm df), FID detector and methanizer. Calibration of GC signals was performed using toluene/CO_2_/air mixtures of known composition. For each temperature at least three separate analyses for substrate and products were repeated. Stability tests were performed by leaving the catalyst at the desired temperature (close to 50% and 90% conversion) and monitoring the changes in activity of the catalyst. 

Conversion of toluene was calculated using Equation (1):

X_tol_ = (C_tol,s_ − C_tol,p_)/C_tol,s_ (1)

where C_tol,s_ and C_tol,p_ are measured concentrations of toluene in the stream at the reactor inlet and outlet. For each temperature, the carbon balance for the gas stream was calculated, showing that CO_2_ is the main product of the toluene combustion. 

#### 2.4.2. O_2_ Temperature-Programmed Desorption (O_2_-TPD)

Oxygen temperature programmed desorption (O_2_-TPD) was performed to gain a better understanding of the oxygen mobile forms within the investigated catalytic systems. Experiments were performed in a quartz U-shape tube reactor in He flow of 40 mL min^−1^. Samples of about 50 mg were heated from room temperature to 800 °C with a heat ramp of 10 °C min^−1^. Prior to the experiment, catalysts were conditioned at 300 °C in 2.5% O_2_/He flow of 40 mL min^−1^ for 1 h.

## 3. Results and Discussion

### 3.1. Materials Characterization

**Figure 1:**
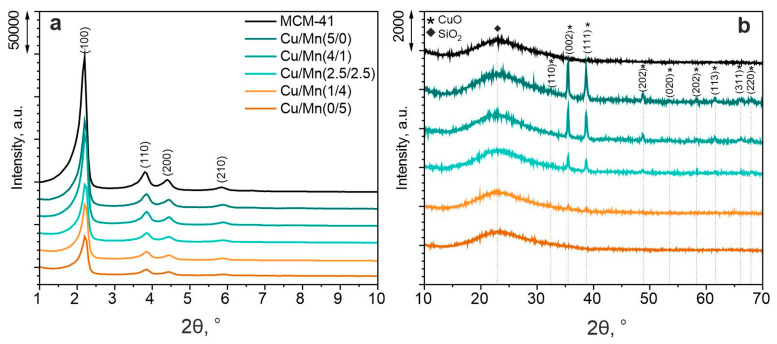
XRD of investigated materials: low-angle reflections (**a**), and wide-angle reflections (**b**).

**Figure 2:**
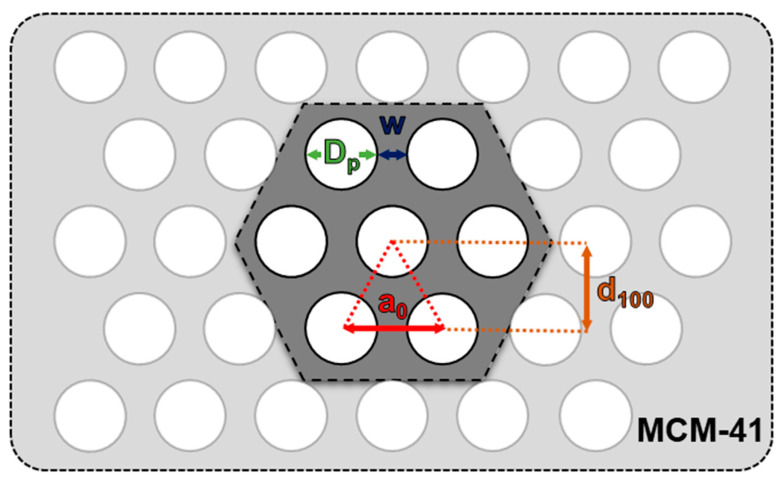
Schematic illustration of MCM-41 and its hexagonal arrangement with identification of (100) plane interplanar distance (d_100_), pore diameter (D_p_), wall thickness (w), and distance between adjacent tube centres (a_0_).

XRD was employed to investigate the phase composition of obtained materials and the results were presented in Figure 1. MCM-41 as an amorphous material does not show reflections in the wide-angle range of the XRD pattern. A broad peak at 2θ of 23° is characteristic of materials containing amorphous silica. However, due to the highly ordered structure of MCM-41, reflections can be observed at low-angle scanning regions of 2θ: 2.20°, 3.80°, 4.40°, and 5.85°, which represent reflective surfaces: (100), (110), (200), and (210), respectively. Their positions also correlated with the JCPDS card number 49-1712, which implies that the highly ordered mesoporous silica material of MCM-41 was successfully obtained in the synthesis using fly ash. These reflections were present for all obtained materials, which proves, that the introduction of metals did not cause the collapse of the MCM-41 structure. Also, from the reflective surface (100), it is possible to calculate the distance between the MCM-41 adjacent tubes (see Figure 2) [19]. For all samples, the calculated d_100_ value was 4.02 nm, which implies the stability of the structure (Table 1). The calculated wall thickness (w) of the obtained materials varied from 1.70 to 1.82 nm. The introduction of metals had a rather negligible effect on the average pore diameter, which varied from 2.82 to 2.94 nm. These values align with those obtained in the literature [20]. However, with an increase in the Mn loading, the intensities of low-angle reflections characteristic of MCM-41 were shrinking. Hence, it might be concluded, that manganese caused pore blockage and was located within the pores or channel mouth of MCM-41, whereas copper was located on the material’s external surface [21]. Such a phenomenon was also reported by Sharma et al. [22]. The authors claimed that the formation of CuO nanocrystallites at MCM-41 occurs on the external surface as the average size of the CuO was >20 nm (compared with ~2.9 nm pore size of MCM-41). In the present study, the average CuO crystal size was about 30 nm (according to the Scherrer equation, not given in the manuscript). There were nine characteristic peaks originating from various CuO crystal planes at the catalyst surface at 2θ: 32.35° (110), 35.50° (002), 38.70° (111), 48.80° (202), 53.40° (020), 58.25° (202), 61.35° (113), 66.15° (311), and 68.00° (220) [23]. The peak positions correlated well with the JCPDS card number 45-0937. Such a wide range of CuO crystal planes and sharp diffraction peaks imply the formation of monoclinic CuO at the material surface [24]. On the other hand, phases of manganese oxides were not detected in the XRD measurement, probably due to its loading being below the detection limit, its high dispersion in the material, or too small crystallite size [21]. Shao et al. [25], have reported that the introduction of Mn species into the MCM-41 structure, causes pore blockage, hence Mn oxides cannot be recorded in the XRD pattern (or due to its low loading within the material). 

The surface properties of the investigated catalysts are summarized in Table 1. It can be seen that the specific surface area (S_BET_) of MCM-41 was decreased after the introduction of metals into the material. Such a phenomenon was evidenced mostly for Cu/Mn(0/5), for which the S_BET_ (744 m^2^ g^−1^) was decreased by about 26% in comparison with parent MCM-41 (981 m^2^ g^−1^). It was also reflected by lower total pore volume (V_t_), hence it might be assumed, that manganese oxide species, according to the literature, occupy the pores causing their partial blockage [21]. For other systems, the S_BET_ was decreased by 9–16%. With a greater loading of Cu, the S_BET_ was higher, implying, that copper occurs on the external surface of MCM-41. However, it can be seen, that regardless of both metal types and their loading to the MCM-41 material, the average pore diameter (D_p_) was maintained and varied from 2.82 to 2.94 nm. There was no significant trend to be observed regarding this variable. 

**Figure 3:**
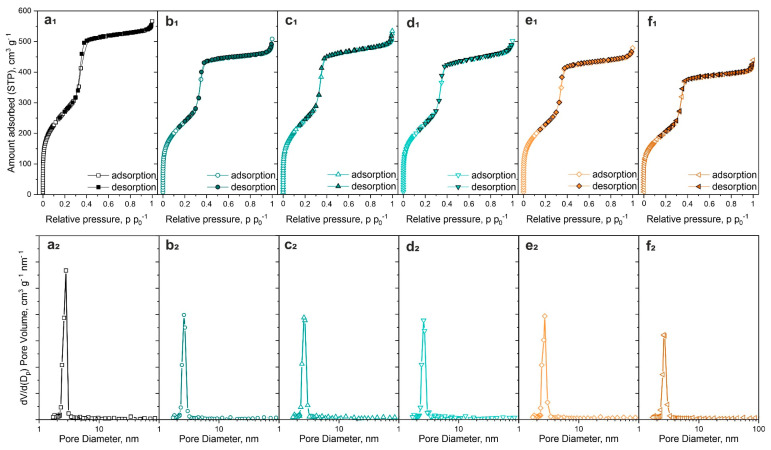
N_2_ adsorption-desorption isotherms at 77K of investigated samples (1), and BJH method adsorption pore distribution (2) of investigated materials: MCM-41 (**a**), Cu/Mn(5/0) (**b**), Cu/Mn(4/1) (**c**), Cu/Mn(2.5/2.5) (**d**), Cu/Mn(1/4) (**e**), Cu/Mn(0/5) (**f**). STP—standard temperature and pressure.

In Figure 3a_1_–f_1_ the N_2_ adsorption-desorption isotherms of investigated samples were presented. According to the IUPAC standard characterization of porous materials and isotherms shapes, the investigated MCM-41 structure displayed a curve shape of IV-A mixed with IV-B. Such a shape is typical for mesoporous materials with smaller width pores (like MCM-41) with a characteristic step in p p_0_^−1^ region of 0.3. [26,27]. The isotherm shape type IV-B also proves the occurrence of conical and cylindrical pores. Small hysteresis loops occurring for samples MCM-41, Cu/Mn(5/0), and Cu/Mn(4/1) were classified as H1 mixed with H2 types. The hysteresis loop was not observed for other samples, for which the Mn loading was greater than 2.5 wt.%. Such observation stays in line with the hypothesis, that manganese oxide species can cause partial pore blockage. Hence, the typical for MCM-41 material hysteresis loop was no longer observed. In Figure 3a_2_–f_2_ the pore size distribution of the MCM-41 materials was presented. It can be seen, that for all investigated samples, there was one dominant pore size of about 2.8 nm, originating from the cylindrical MCM-41 pores of uniform distribution. Moreover, the values obtained from N_2_ adsorption-desorption experiments are consistent with those from XRD calculations, implying that the modification to the MCM-41 did not cause the destruction of the material. The obtained results remain in line with the literature reports [25,28]. 

The chemical composition of investigated materials was examined by means of two independent methods: XRF and EDS. The results are summarized in Table 2. It can be seen, that both measurements indicated familiar chemical composition of samples, with O, Si, Cu, and Mn as the main components. The theoretical Cu and Mn loadings remain in line with the obtained results and prove the successful introduction of metal species into the MCM-41 matrix, as can be seen from both XRF and EDS measurements. The additional elements occurring within the materials were Al and Mg, which may be the residue originating from fly ash [9]. However their contents were significantly below 2 wt.%.

**Figure 4:**
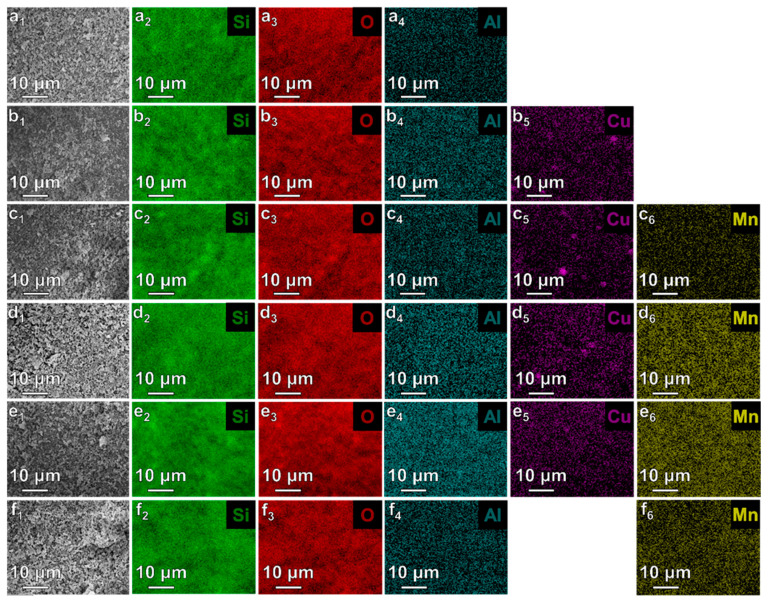
SEM images (1), and EDS mapping (2–6) of investigated samples: MCM-41 (**a**), Cu/Mn(5/0) (**b**), Cu/Mn(4/1) (**c**), Cu/Mn(2.5/2.5) (**d**), Cu/Mn(1/4) (**e**), Cu/Mn(0/5) (**f**).

In Figure 4 the SEM images of investigated samples and EDS mapping are compared. It can be seen that the following elements were identified (Si, O, Cu, Mn) over the MCM-41 and modified MCM-41 sample surfaces. For samples containing higher loadings of Cu i.e., Cu/Mn(5/0), and Cu/Mn(4/1), there were agglomerates of Cu occurring over the surface. However, the introduced metals appeared to be well dispersed. Segregation of Cu species over the catalyst surface, might be related to the fact, that Cu occurs on the external surface of the material, whereas Mn might be located in pores of MCM-41, as was described above. On the other hand, in the research introduced by Deshmane et al. [29], it was reported, that Cu tends to create agglomerates on MCM-41 surface when the metal loadings exceed 10 wt.%.

**Figure 5:**
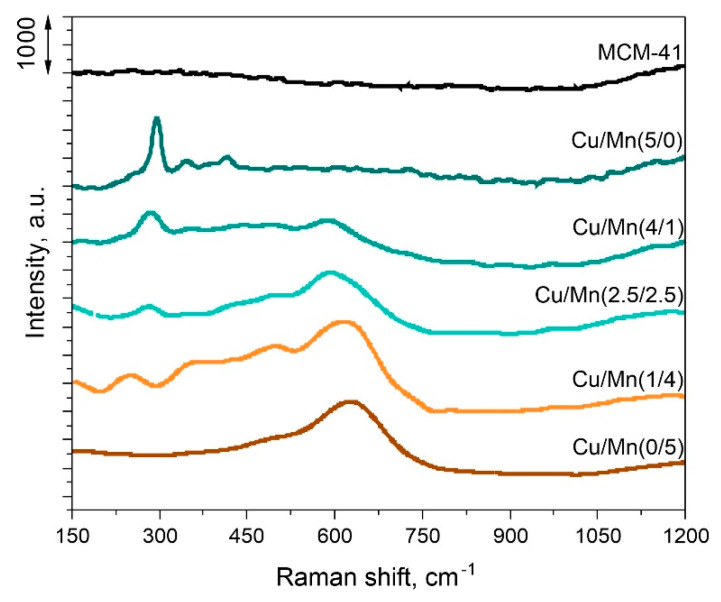
Raman spectra of investigated samples.

The Raman spectroscopy was employed to investigate the structure of investigated catalysts. Recorded spectra are presented in Figure 5. It can be seen, that parent MCM-41 shows no vibrations with this technique. The occurrence of Cu and Mn bonds was confirmed. Vibrations from CuO were clearly identified at around 296 (A_g_), 348 (B_g_), and 417 cm^−1^ [30]. Their intensities significantly decreased as the loading of Cu was lowered. A broad peak occurring for all samples containing Mn with a maximum at around 630 cm^−1^ was assigned to MnO_x_ species over the MCM-41 surface. It can also be seen that the peak maximum shifted from lower (594 cm^−1^) to higher values (619 cm^−1^) as the Mn loading increased from 1 to 4 wt.%. This phenomenon might imply interactions occurring between Cu and Mn species, causing shift in the maximum peak intensity [31]. 

**Figure 6:**
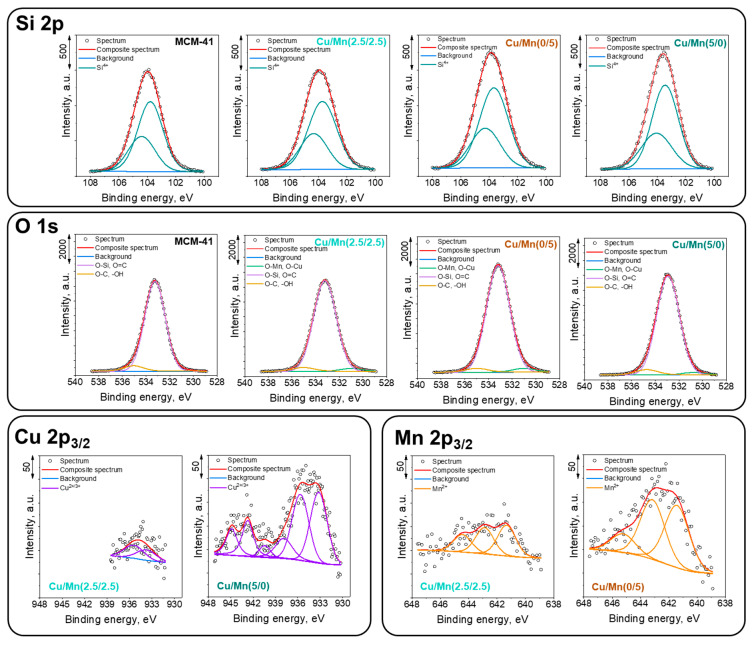
XPS spectra of selected samples: MCM-41, Cu/Mn(5/0), Cu/Mn(2.5/2.5), and Cu/Mn(0/5).

The XPS spectra were recorded to gain a better knowledge of the surface chemistry of the investigated catalysts and the occurrence of different species according to the differences in the binding energies. Four samples were selected for the measurement: MCM-41, Cu/Mn(5/0), Cu/Mn(2.5/2.5), and Cu/Mn(0/5). The obtained results are summarized in Figure 6 and in Table 3. 

The Si 2p spectra show one doublet structure (doublet separation p_3/2_–p_1/2_ equals 0.6 eV) with the main 2p_3/2_ line centered at 103.7 eV which indicates the Si^4+^ oxidation state of silicon in compounds such as silicates [32,33].

The O 1s spectra were fitted with three components: the first line centered at 531.0 eV which points out to the existence of metal oxides (O-Cu, O-Mn); the second line centered at 533.2 eV, which indicates the presence of mainly O-Si-type bonds and either defective oxygen in metal oxides or organic species (O=C); and the last line found at 535.0 eV which originates from either -OH and/or C-O-type bonds from organic contamination and/or adsorbed water [32,34]. It can be clearly seen, that the introduction of metal species promoted occurrence of oxygen species over the catalysts surface (line at 531.0 eV). As the lattice oxygen is reported to have a beneficial role in VOC oxidation, it can be noted, that its greatest share was obtained for Cu/Mn(0/5) and Cu/Mn(2.5/2.5) samples. Simultaneously, the share of O-Si-type bonds diminished as the Mn loading increased.

Spectra collected at the Cu 2p_3/2_ region were fitted with up to six components with the first line centered at 933.3 eV, which indicates the existence of the Cu^2+/3+^ oxidation states [34,35]. Three lines within the energy range of 940–945 eV are due to the shake-up processes mostly intense only in the case of the Cu^2+/3+^ oxidation states presence. This is consistent with the XRD results (Figure 1b), where CuO was confirmed to be present over the catalyst surface.

Spectra collected at Mn 2p_3/2_ region were fitted with three components with the first line centered at 640.2 eV which points out the existence of the Mn^2+^ oxidation state. The two lines within the energy range of 640–644 eV are due to the multiplet splitting phenomena and the position of last shake-up line found at ~645 eV is additional parameter which ensures the Mn^2+^ oxidation state of manganese in the samples [34,35]. Occurrence of two regions characteristic of Mn^3+^ and Mn^4+^ can be found at about 641 and 644 eV, respectively [36]. It was evidenced, that Mn^3+^ species can be responsible for oxygen transfer between the catalyst and VOCs, whereas Mn^4+^ species serve as oxygen reservoirs [37]. 

### 3.2. Catalytic Activity of Materials

#### 3.2.1. Catalytic Combustion of Toluene

**Figure 7:**
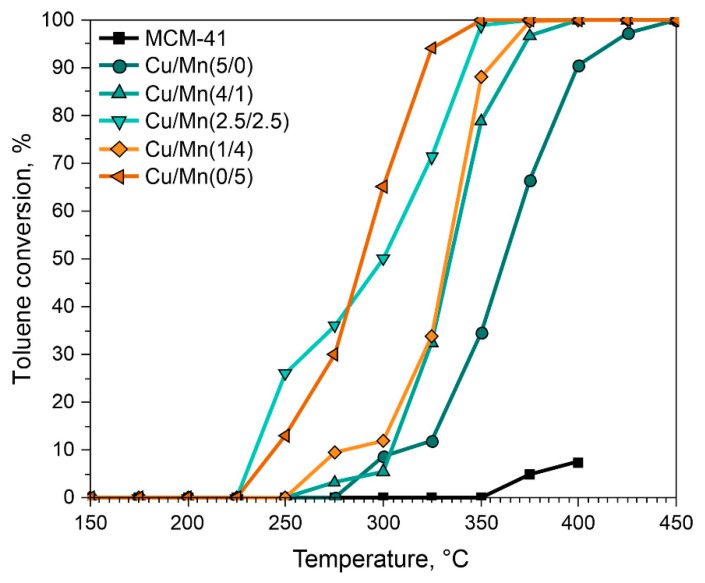
Catalytic combustion of toluene over MCM-41 catalysts.

MCM-41 derived from coal fly ash (CFA) catalysts supported with Cu and/or Mn were evaluated in the catalytic combustion of toluene. The results of catalytic tests are summarized in Figure 7. The highest catalytic activity was found for Cu/Mn(0/5) and Cu/Mn(2.5/2.5). The measured values of T_50_ (temperature of 50% conversion) were found to be 287 and 300 °C, respectively. Catalysts Cu/Mn(1/4) and Cu/Mn(4/1) demonstrated comparable activity through the entire catalytic test (T_50_ of 325 °C). For Cu/Mn(5/0), the activity was the lowest among all investigated catalyst series and the T_50_ was 360 °C. For the parent MCM-41 material, the catalytic test was conducted up to 400 °C as there were no significant changes in the activity and the maximum conversion at this temperature was about 7.5%. As for the ignition curve, it varied depending on the metal loading and type of metal introduced. The most rapid ignition was detected for Cu/Mn(2.5/2.5) and Cu/Mn(0/5). Hence, manganese favors, to the a greatest extent the combustion of toluene. It should be stated, that the specific surface area does not play a crucial role in the investigated process as parent MCM-41 and Cu/Mn(5/0) exhibited the highest S_BET_ (Table 1) and lowest catalytic activity. Such a phenomenon was also reported in the work of Bialas et al. [38]. The most active Cu/Mn(0/5) catalyst possesses the lowest surface area among studied samples due to pore blocking by MnO_x_ particles. The second most active catalyst Cu/Mn(2.5/2.5) clearly shows, that this composition of active phase is beneficial, as it possesses a lower concentration of Mn than Cu/Mn(1/4) and similar S_BET_. This result confirms the promotional role of copper in the studied catalyst. Such a phenomenon was evidenced in the work of Kaewbuddee et al. [39], where Cu addition to cryptomelane (K-OMS-2) structure was reported to improve oxygen mobility, which can favor toluene catalytic oxidation and its more rapid ignition.

It was reported that the transfer of oxygen and oxygen vacancies promotes the combustion of toluene [36]. Both adsorbed and lattice oxygen can play an essential role in the so-called Mars-van Krevelen mechanism [40]. The activity of Mn containing catalyst was well described by Zhang et al. [41], where the authors implied the promotional role of Mn^3+^ species and surface oxygen species on toluene oxidation. Various MnO_x_ structures were intensively studied in recent years, due to their strong oxidizability, i.e., rods, tubs, sphere, 3DOM (three-dimensionally ordered microporous materials), and, most importantly, due to this feature–a share of mobile lattice oxygen species [42]. Therefore, Cu/Mn(0/5) was characterized by the greatest catalytic performance in the examined reaction. The promotional effect of Cu addition was clearly observed when the relative ratio of Cu/Mn was 2.5/2.5 and was reflected in the lowest ignition temperature among the investigated catalyst series. It might be related to the optimal share of oxygen-metal bonds present over the material’s surface, as was evidenced in the XPS analysis (Figure 6 and Table 3).

**Figure 8:**
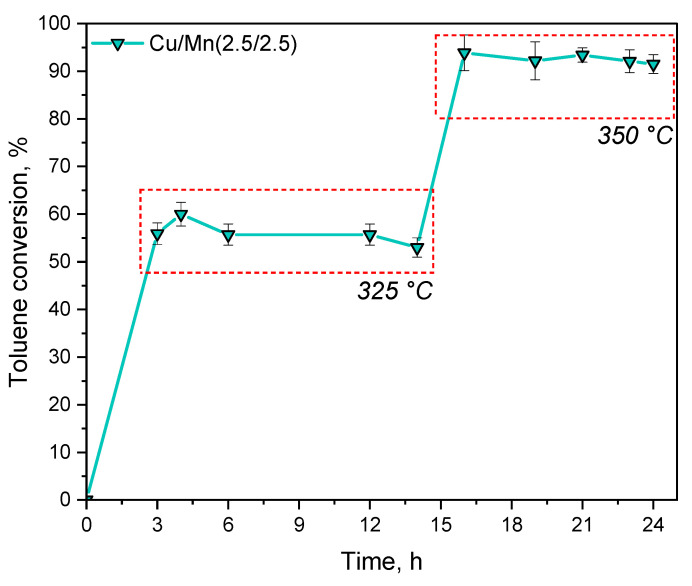
Time-on-stream combustion of toluene.

The time-on-stream test was conducted to investigate the stability of the Cu/Mn(2.5/2.5) catalyst in toluene combustion reaction. The results are summarized in Figure 8. The test was conducted in two regimes, namely at 325 °C (for 14 h) and at 350 °C (for 10 h). The fluctuations in toluene conversion at a temperature of 325 °C can be a result of overactivity of the Cu/Mn(2.5/2.5) at the beginning of the process. However, these fluctuations were rather insignificant through the catalytic test. The catalysts appeared to be stable in the selected temperature windows and the drop in toluene conversion was rather negligible (especially at 350 °C). Moreover, the obtained toluene conversion values were comparable to those obtained from the initial catalytic test as can be seen in Figure 7.

#### 3.2.2. O_2_-TPD

**Figure 9:**
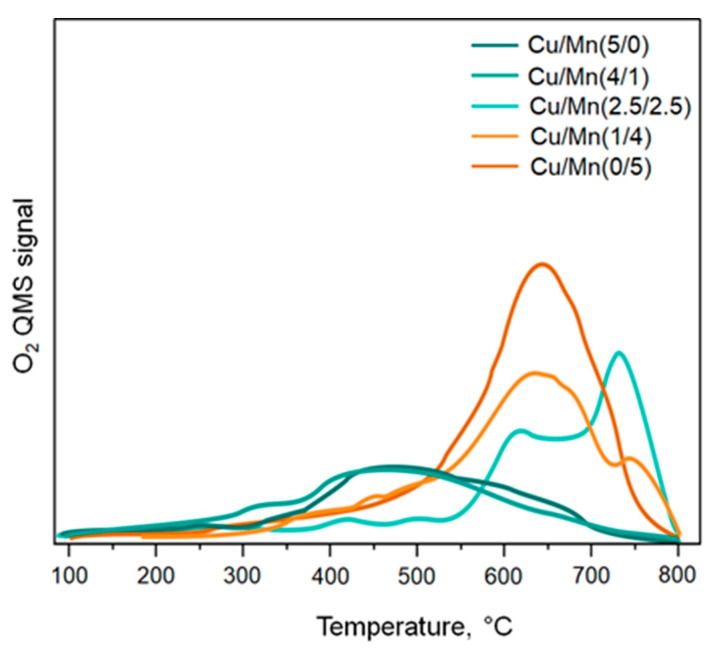
O_2_-TPD profiles of investigated catalysts.

The redox properties of investigated catalysts were examined by means of oxygen temperature-programmed desorption (O_2_-TPD). The results are presented in Figure 9. It was reported, that oxygen released <400 °C and >400 °C was assigned to adsorbed oxygen (O_ads_) and lattice oxygen (O_latt_), respectively. More precisely, the release of oxygen in the range <400 °C is attributed to the atomic-state oxygen (O_2_^−^, O^−^) adsorbed over the metal oxides. Oxygen released >400 °C was assigned as lattice oxygen species (O^2−^) and appeared from 1 to 3 maxima depending on the metal type and loading [37]. The first type of oxygen species that appeared from 400 to 500 °C was a broad peak labeled α. It was the most dominant for Cu/Mn(5/0) and Cu/Mn(4/1). An intensive peak with a maximum of about 650 °C was detected for Cu/Mn(0/5), and Cu/Mn(1/4) catalysts and labeled β. The last peak γ detected at high temperatures of about 740 °C was detected for Cu/Mn(2.5/2.5) and Cu/Mn(1/4) implying strong interaction between Mn and Cu and a high share of lattice oxygen. A familiar phenomenon was observed by Zhao et al. [42], where the addition of a small amount of ceria to the catalyst, resulted in a significant shift in the O_2_-TPD profile at high temperatures (about 800 °C), implying strong interaction between these metals. Such an interaction can bring a promotive effect in the catalytic oxidation process. It can be also concluded that the peak locations and intensities of oxygen released from the samples strictly depended upon the metal introduced and its loading [43]. It should be noted, that the oxygen species occurring in the toluene combustion temperature window, were only the O_ads_ species, whose share was relatively low for all samples. However, the experiment clearly evidenced the occurrence of Cu/Mn interactions, which were the most seen for Cu/Mn(2.5/2.5), allowing the catalyst to demonstrate the most rapid ignition among the investigated series.

## 4. Conclusions

In the present study, MCM-41 from fly ash was obtained and evaluated as a catalyst support for toluene combustion. MCM-41 structure was successfully synthesized from silica reach solution after zeolite synthesis from fly ash. Hence, in the light of the environmental protection, the potential pathway of fly ashes utilization was confirmed. It was evidenced, that the structure of MCM-41 was maintained after the introduction of metal phases (Cu/Mn) and varying loadings and thermal treatment. The catalytic combustion of toluene allowed us to identify the optimal Cu/Mn loading of 2.5/2.5 wt.%. It was reflected in the lowest loading of each of the metals and the lowest ignition temperature resulting from Cu-Mn interaction and share of oxygen species. It was evidenced, that the specific surface area was not the crucial parameter affecting the catalytic activity. The time-on-stream test was conducted to demonstrate the thermal stability of the examined Cu/Mn(2.5/2.5) catalytic system. In a 24 h test conducted at 325 and 350 °C it was found that, the conversion of toluene was stable throughout the test (as observed at 350 °C). MCM-41 mesoporous silicate from fly ash can serve as a promising and efficient catalyst support in VOC oxidation reactions.

## Figures and Tables

**Table 1:** Surface properties of obtained materials.

Sample	S_BET_, m^2^ g^−1^	V_t_, cm^3^ g^−1^	d_100_, nm	a_0_, nm	D_p_, nm	w, nm
MCM-41	981	0.84	4.02	4.64	2.86	1.78
Cu/Mn(5/0)	904	0.78	4.02	4.64	2.82	1.82
Cu/Mn(4/1)	891	0.77	4.02	4.64	2.86	1.78
Cu/Mn(2.5/2.5)	840	0.74	4.02	4.64	2.94	1.70
Cu/Mn(1/4)	826	0.71	4.02	4.64	2.88	1.76
Cu/Mn(0/5)	744	0.64	4.02	4.64	2.84	1.80

S_BET_—specific surface area; V_t_—total pore volume; d_100_—(100) plane interplanar distance, d_100_ = n λ/(2sin(θ_100_)), where n = 1, λ = 0.15418 nm, and θ_100_ = 1.1; d: a_0_—distance between adjacent tube centres, a_0_ = 2d_100_/$\sqrt{3}$; D_p_—average pore diameter obtained from BJH method; w—wall thickness, w = a_0_ − D_p_.

**Table 2:** Comparison of chemical composition of investigated materials as obtained from XRF and EDS methods.

	Sample					
Element	MCM-41	Cu/Mn(5/0)	Cu/Mn(4/1)	Cu/Mn(2.5/2.5)	Cu/Mn(1/4)	Cu/Mn(0/5)
Chemical composition as obtained from XRF, wt.%						
Si	45.76 ± 0.31	42.80 ± 0.24	44.07 ± 0.33	43.37 ± 0.11	44.19 ± 0.29	44.09 ± 0.19
Mn	-	-	1.12 ± 0.03	2.28 ± 0.08	4.07 ± 0.08	4.93 ± 0.09
Cu	-	4.94 ± 0.01	4.29 ± 0.05	2.52 ± 0.00	0.85 ± 0.02	-
Mg	1.26 ± 0.10	1.03 ± 0.08	1.31 ± 0.00	1.13 ± 0.02	-	-
Chemical composition as obtained from EDS, wt.%						
O	62.9 ± 0.2	56.8 ± 0.2	62.2 ± 0.2	60.4 ± 0.3	61.0 ± 0.1	55.4 ± 0.1
Si	35.6 ± 0.1	36.9 ± 0.3	31.9 ± 0.3	33.0 ± 0.1	32.2 ± 0.2	36.7 ± 0.2
Al	1.4 ± 0.1	1.6 ± 0.1	1.7 ± 0.1	1.2 ± 0.1	1.7 ± 0.1	1.8 ± 0.2
Cu	-	4.7 ± 0.2	3.4 ± 0.2	2.9 ± 0.1	1.2 ± 0.1	-
Mn	-	-	0.9 ± 0.2	2.6 ± 0.1	3.9 ± 0.1	6.1 ± 0.3

**Table 3:** Surface composition (atomic %) determined by fitting XPS spectra for MCM-41, Cu/Mn(5/0), Cu/Mn(2.5/2.5), and Cu/Mn(0/5).

Element	C			O			Si	Mn	Cu
Binding Energy, eV	285.0	286.2	289.2	531.0	533.2	535.0	102.7	640.2	933.3
MCM-41	6.7	1.2	0.3	1.2	57.4	4.0	29.2	0	0
Cu/Mn(5/0)	1.2	0.2	0	1.5	62.0	3.7	30.3	0	1.1
Cu/Mn(2.5/2.5)	6.9	1.7	0.7	1.8	57.4	3.0	27.9	0.4	0.1
Cu/Mn(0/5)	5.8	2.5	0.6	2.1	58.2	2.5	27.5	0.8	0

## Data Availability

Data are contained within the article.
